# Histone H3K27 demethylase UTX compromises articular chondrocyte anabolism and aggravates osteoarthritic degeneration

**DOI:** 10.1038/s41419-022-04985-5

**Published:** 2022-06-08

**Authors:** Wei-Shiung Lian, Re-Wen Wu, Jih-Yang Ko, Yu-Shan Chen, Shao-Yu Wang, Chun-Ping Yu, Holger Jahr, Feng-Sheng Wang

**Affiliations:** 1 Core Laboratory for Phenomics and Diagnostics, Kaohsiung Chang Gung Memorial Hospital and Chang Gung University College of Medicine, Kaohsiung, Taiwan; 2 Center for Mitochondrial Research and Medicine, Kaohsiung Chang Gung Memorial Hospital and Chang Gung University College of Medicine, Kaohsiung, Taiwan; 3 Department of Medical Research, Kaohsiung Chang Gung Memorial Hospital and Chang Gung University College of Medicine, Kaohsiung, Taiwan; 4 Department of Orthopedic Surgery, Kaohsiung Chang Gung Memorial Hospital and Chang Gung University College of Medicine, Kaohsiung, Taiwan; 5 Biodiversity Research Center, Academia Sinica, Taipei, Taiwan; 6 Department of Anatomy and Cell Biology, University Hospital RWTH Aachen, Aachen, Germany; 7 Department of Orthopedic Surgery, Maastricht University Medical Center, Maastricht, The Netherlands

**Keywords:** Osteoarthritis, Experimental models of disease

## Abstract

Epigenome alteration in chondrocytes correlates with osteoarthritis (OA) development. H3K27me3 demethylase UTX regulates tissue homeostasis and deterioration, while its role was not yet studied in articulating joint tissue in situ. We now uncovered that increased UTX and H3K27me3 expression in articular chondrocytes positively correlated with human knee OA. Forced UTX expression upregulated the H3K27me3 enrichment at transcription factor Sox9 promoter, inhibiting key extracellular matrix molecules collagen II, aggrecan, and glycosaminoglycan in articular chondrocytes. Utx overexpression in knee joints aggravated the signs of OA, including articular cartilage damage, synovitis, osteophyte formation, and subchondral bone loss in mice. Chondrocyte-specific Utx knockout mice developed thicker articular cartilage than wild-type mice and showed few gonarthrotic symptoms during destabilized medial meniscus- and collagenase-induced joint injury. In vitro, Utx loss changed H3K27me3-binding epigenomic landscapes, which contributed to mitochondrial activity, cellular senescence, and cartilage development. Insulin-like growth factor 2 (Igf2) and polycomb repressive complex 2 (PRC2) core components Eed and Suz12 were, among others, functional target genes of Utx. Specifically, Utx deletion promoted Tfam transcription, mitochondrial respiration, ATP production and Igf2 transcription but inhibited Eed and Suz12 expression. Igf2 blockade or forced Eed or Suz12 expression increased H3K27 trimethylation and H3K27me3 enrichment at Sox9 promoter, compromising Utx loss-induced extracellular matrix overproduction. Taken together, UTX repressed articular chondrocytic activity, accelerating cartilage loss during OA. Utx loss promoted cartilage integrity through epigenetic stimulation of mitochondrial biogenesis and Igf2 transcription. This study highlighted a novel noncanonical role of Utx, in concert with PRC2 core components, in controlling H3K27 trimethylation and articular chondrocyte anabolism and OA development.

## Introduction

Osteoarthritis (OA) is the most common form of arthritis, developing a degenerative loss of articular cartilage [1]. Erosion of the cartilage extracellular matrix (ECM) causes a plethora of osteoarthritic symptoms, including synovial swelling, osteophyte formation, and subchondral plate sclerosis [1]. Expanding evidence suggests that dysregulated expression of, among others, chondrocyte key transcription factors [2], canonical Wnt signaling components [3], and matrix catabolic factors [4, 5] induce chondrocyte dysfunction which in turn accelerates OA development. The underlying mechanisms leading to this change in metabolic activity in osteoarthritic chondrocytes remains, however, poorly elucidated.

Epigenetic pathways chemically modify DNA-bound histones, controlling promoter activities for gene transcription [6]. Histone methylation enhances chromatin condensation, which silences gene expression and thus changes cellular activities [6]. For example, the hypomethylation of lysine-36 in histone 3 (H3K36) correlates with chondroblastoma [7]. H3K9 methylation represses SOX9 transcription, slowing chondrogenesis and skeletal morphogenesis in mice deficient in AT-rich interactive domain 5b [8]. Mice deficient in disruptor of telomeric silencing 1-like, one of H3K36 methyltransferases, in chondrocytes have defective skeletal phenotypes [9] and spontaneous OA development [10]. Moreover, methylated H3K4 involves the knee OA changes in aged mice and is further relevant to human OA [11].

Histone demethylase UTX removes the trimethyl group from K27 in histone H3 (H3K27me3). In contrast, histone methyltransferase polycomb repressive complex 2 (PRC2) core components, including enhancer of zeste homolog 2 (EZH2), embryonic ectoderm development (EED), and PRC2 subunit (SUZ12), catalyze trimethylation of H3K27 [12]. While UTX generally promotes gene activation and appears essential during normal development and tissue-specific differentiation [13], increased H3K27me3 in cartilage correlates with human hip OA [14]. Mice lacking Ezh1 and Ezh2 in chondrocytes show skeletal tissue underdevelopment and decreased H3K27me3 abundances [15]. Ezh2 deletion accelerates the development of OA in mice [16], while chondrocyte-specific Eed knockout mice show a deformed skeleton and a decrease in chondrocyte survival [17]. The role of UTX during articular cartilage homeostasis and progression of OA remains poorly understood.

This study aimed at using human OA specimens, UTX knockdown and overexpression in chondrocytes, and chondrocyte-specific UTX knockout mice to study how UTX altered the articular cartilage phenotype or OA development and how it affected H3K27 trimethylation and epigenomic landscapes, which contributed to chondrocytic activity.

## Results

### UTX and H3K27me3 correlated with human gonarthrosis

**Fig. 1:**
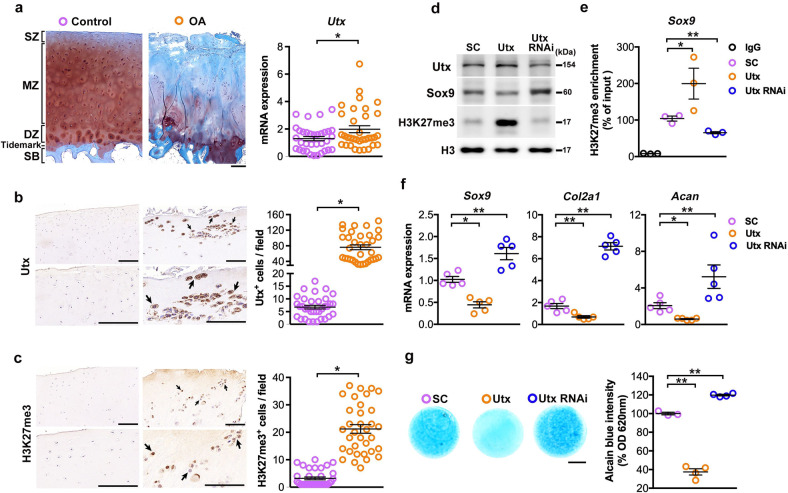
UTX-dependent reduction in chondrocytic activities. Safranin-O staining of macroscopically normal human articular cartilage next to severely osteoarthritic tissue. Quantification of UTX mRNA abundance in both tissues revealed elevated UTX expression in gonarthrotic cartilage (**a**); scale bar, 200 μm. Strong UTX (**b**) and H3K27me3 (**c**) immunostaining in osteoarthritic chondrocytes, next to quantified protein expression from 34 donors; scale bar, 20 μm (low magnification), 10 μm (high magnification). Forced UTX expression increased H3K27me3 levels and reduced Sox9 abundance in articular chondrocytes (**d**). Utx gain-of-function upregulated H3K27me3 enrichment at the Sox9 promoter (**e**), but reduced *Sox9*, *Col2a1* and *Acan* expression (**f**), respectively, and glycosaminoglycan synthesis (**g**) quantified by Alcian blue staining; scale bar, 500 μm. Utx knockdown repressed H3K27me3 levels and promoted chondrocytic activity. Cells were transfected with Utx RNAi or cDNA or scramble control for 24 hours. RT-PCR was conducted upon transfection for 24 h. Micromass for Alcian blue staining were incubated for 7 days. Culture experiments were conducted from three to five mice and data are expressed as mean ± standard error; *P < 0.05; **P < 0.001. SC scrambled control.

UTX Safranin-O-stained sections showed typical histopathology alterations and increased UTX mRNA expression in human OA cartilage, as compared to macroscopically healthy cartilage, lateral to the injured site in patients with end-stage knee OA (Fig. 1a). Osteoarthritic chondrocytes exhibited strong UTX (Fig. 1b) and H3K27me3 (Fig. 1c) immunostaining, respectively, confirming this on protein level.

### UTX inhibited ECM production in chondrocytes

Next, we investigated Utx expression in relation to ECM expression in articular chondrocytes. Chondrocytes from knee joints of 7-day-old mice were transfected with Utx expressing vectors or Utx siRNAs, respectively. Forced Utx expression increased H3K27me3 levels but decreased Sox9 abundance (Fig. 1d). It promoted H3K27me3 enrichment at the Sox9 promoter (Fig. 1e) and reduced expression of Sox9 and cartilage-specific key ECM markers Col2a1 and Acan (Fig. 1f). Consequently, Alcian blue staining confirmed ECM loss in micromass cultures (Fig. 1g). Silencing Utx expression reduced overall H3K27me3 levels (Fig. 1d) and its occupancy at the Sox9 promoter (Fig. 1e), but enhanced expression of chondrocyte markers (Fig. 1f) and ECM production (Fig. 1g).

### Forced Utx expression caused OA-like symptoms

**Fig. 2:**
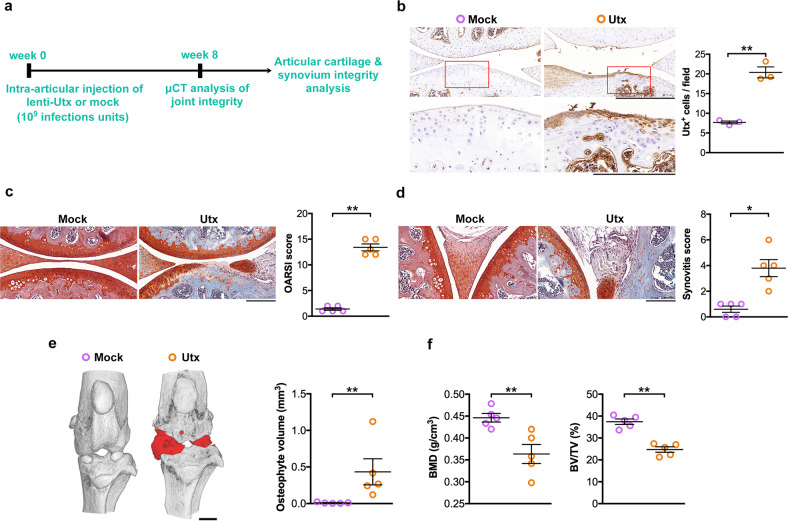
Signs of OA upon forced expression of Utx in mice. Schematic drawing for intra-articular injection of lentivirus *Utx* (**a**). Strong Utx immunostaining in articular cartilage upon intra-articular injection with lentiviruses expressing *Utx* (**b**); scale bar, 20 μm (low magnification) and 10 μm (high magnification). Severe cartilage disintegration (**c**; scale bar, 20 μm) and synovial hyperplasia (**d**; scale bar, 100 μm) in Utx-treated murine knees, as evident from OARSI and synovitis scoring. Forced Utx expression induced osteophyte formation (red; scale bar, 0.5 mm) (**e**) but reduced BMD and BV/TV (**f**) of subchondral bone. Data are expressed as mean ± standard errors (*n* = 5). *P< 0.05; **P < 0.001.

Utx-mediated chondrocyte dysfunction in vitro prompted us to investigate whether increasing Utx expression affected the integrity of diarthrodial joints. Tibiofemoral compartments were intra-articularly injected to lentivirally overexpress *Utx* (Fig. 2a). Subsequently, articular chondrocytes displayed strong Utx immunostaining (Fig. 2b). Forced Utx expression further induced signs of severe OA development, including cartilage degeneration (Fig. 2c) and synovial hyperplasia (Fig. 2d) together with increases in OARSI- and synovitis scores. Furthermore, μCT images showed evident osteophyte formation around the injured knees and increased osteophyte volume (Fig. 2e), while BMD and BV/TV of subchondral compartment were decreased (Fig. 2f) through Utx overexpression.

### Chondrocyte-specific Utx knockout protected articular cartilage integrity

**Fig. 3:**
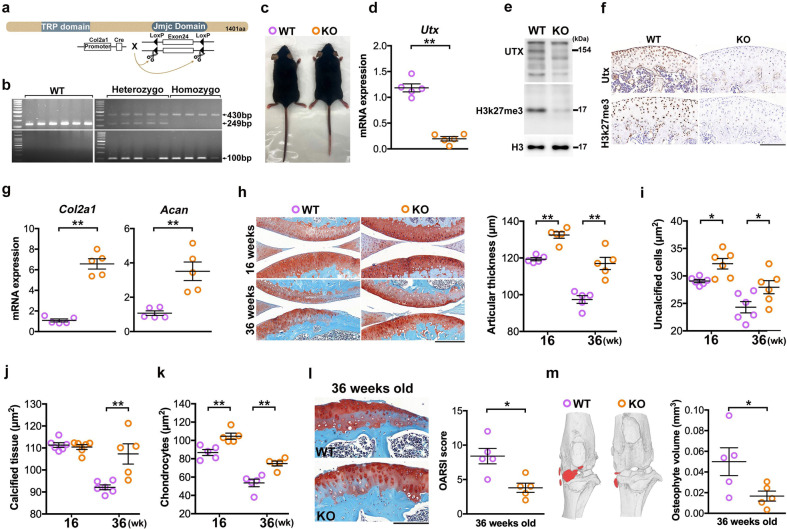
Utx knockout promoted ECM synthesis and protected articular cartilage integrity. Schematic drawing of the generation of chondrocyte-specific Utx knockout mice; exon 24 of Utx, containing the JmjC domain of the enzyme, was floxed and cleavage of these sites resulted in the transcription/translation of inactive Utx. Through mating Utx mice with Col2-Cre mice, its expression is further under control of the cartilage-specific collagen type II gene promoter (**a**). Confirmation of proper genotypes of UtxKO and WT mice; heterozygous UtxKO mice carried flox constructs corresponding to 430 and 249 bp, whereas homozygous UtxKO mice only expressed amplicons corresponding to 430 bp. Absence of a 100 bp PCR amplicon in WT mice, corresponding to the Cre construct, but presence in UtxKO mice (**b**). Appearance and hair color of UtxKO mice were similar to WT mice (**c**). mRNA expression (**d**) and protein abundance (**e**), respectively, confirming the absence of Utx and reduced H3K27me3 levels in UtxKO mice. Very faint Utx and H3K27me3 immunostaining in articular cartilage of KO mice (**f**); scale bar, 10 μm. Utx loss promoted Col2a1 and Acan expression (**g**). Safranin-O staining of articular cartilage (**h**; scale bar, 100 μm) and increased relative thickness of articular cartilage, uncalcified portion (**i**), calcified zone (**j**), and density of articular chondrocytes (**k**), respectively, in UtxKO mice. Articular cartilage degeneration and decreased OARSI scores (**l**) together with osteophyte formation (arrows) in 9-month-old mice (**m**). Data are expressed as mean ± standard errors calculated from five to six mice. *P < 0.05; **P < 0.001.

As Utx knockdown enhanced expression of chondrocyte differentiation markers in vitro, we generated chondrocyte-specific UtxKO mice. The Utx flox was flanked exon 24 (Fig. 3a), which is indispensable for its demethylase activity [18]. Genotyping subsequently confirmed Utx loss (Fig. 3b, c) and cartilage-specific absence of Utx expression in these animals (Supplementary Fig. S1). Birth frequency of homozygous UtxKO mice was similar to the expected Mendelian frequency (Supplementary Fig. S1). Upon confirmation of absence of Utx mRNA (Fig. 3d) and protein expression, respectively, H3K27me3 levels were reduced in chondrocytes (Fig. 3e). Consistent with earlier in vitro analyses, articular UtxKO chondrocytes did not show Utx, or convincing H3K27me3, immunostaining (Fig. 3f), while cartilage key ECM markers Col2a1 and Acan expression were increased (Fig. 3g).

UtxKO mice have relatively thicker articular cartilage (Fig. 3h) and uncalcified portion (Fig. 3i) than WT animals at both tested timepoints; however, the calcified zone was unaffected (Fig. 3j). Nine-month-old animals in both groups showed thinning of the articular cartilage, with that of UtxKO mice remaining relatively thicker than that of WT animals. While the number of articular chondrocytes declined in both groups (Fig. 3k), their numbers stayed always relatively higher in UtxKO animals. Articular cartilage erosion (Fig. 3l) and osteophyte formation (Fig. 3m) were evident in joints at 9 months old. Overall, UtxKO mice showed better articular cartilage with lower OARSI scores and a reduction in osteophyte volume.

### UTX knockout delayed OA development

**Fig. 4:**
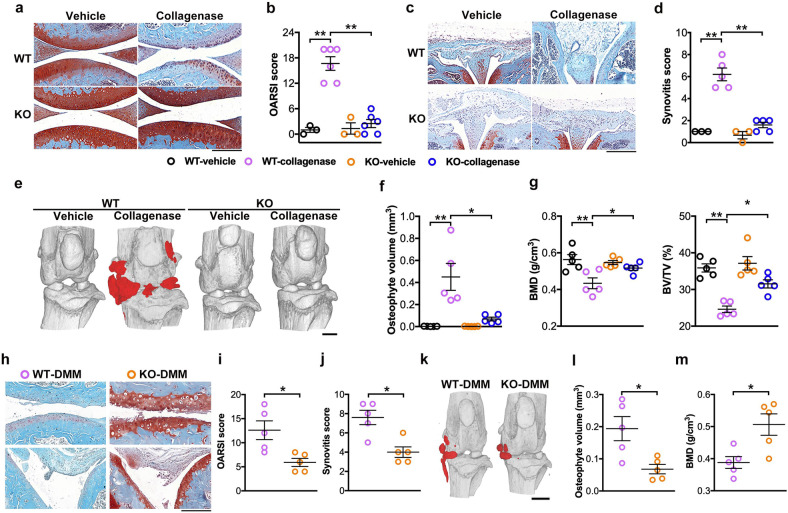
UtxKO mice showed few signs of collagenase or destabilized medial meniscus-induced gonarthrosis. Upon OA induction, UtxKO mice developed weaker symptoms, including articular cartilage degradation (**a**; scale bar, 20 μm) and quantitative OARSI scores (**b**) together with synovitis (**c**; scale bar, 100 μm) and synovitis scores (**d**). Utx loss compromised osteophyte formation (**e** scale bar, 500 μm), osteophyte volume **(f**) and loss in subchondral BMD and BV/TV (**g**) of UtxKO mice. Eight weeks DMM postoperatively, UtxKO mice showed less cartilage damage and synovitis (**h** scale bar, 20 μm) together with decreased OARSI scores (**i**) and synovitis scores (**j**) than WT mice. Osteophyte formation (**k**), osteophyte volume (**l**), and subchondral bone BMD (**m**) were compromised in UtxKO mice. Data are expressed as mean ± standard errors calculated from five to six mice. *P < 0.05; **P < 0.001.

Given that high Utx expression was associated with human knee OA (Fig. 1) and articular cartilage degeneration in mice (Fig. 2), we used intra-articular collagenase injections [19] and DMM [20] as two accepted models to induce OA development. Eight weeks upon collagenase injection, joints in WT mice showed severe cartilage loss (Fig. 4a) and synovial hyperplasia (Fig. 4c) together with increased OARSI- (Fig. 4b) and synovitis scores (Fig. 4d), respectively. Osteophytes were more prominently developed in WT than in UtxKO animals (Fig. 4e), revealing increased overall osteophyte volumes (Fig. 4f) and decreased subchondral BMD and BV/TV (Fig. 4g) by μCT analysis. Strikingly, cartilage destruction, synovitis, osteophyte formation, and subchondral bone mass loss were all compromised in UtxKO mice.

Eight weeks upon DMM induction, articular cartilage loss and synovial hyperplasia (Fig. 4h) together with OARSI scores (Fig. 4i) and synovitis scores (Fig. 4j) in UtxKO mice were less than in WT mice. Osteophyte formation (Fig. 4k, l) and subchondral bone mass loss (Fig. 4m) were repressed in UtxKO animals.

### UTX loss decreases H3K27me3 enrichment at mitochondrial genes

**Fig. 5:**
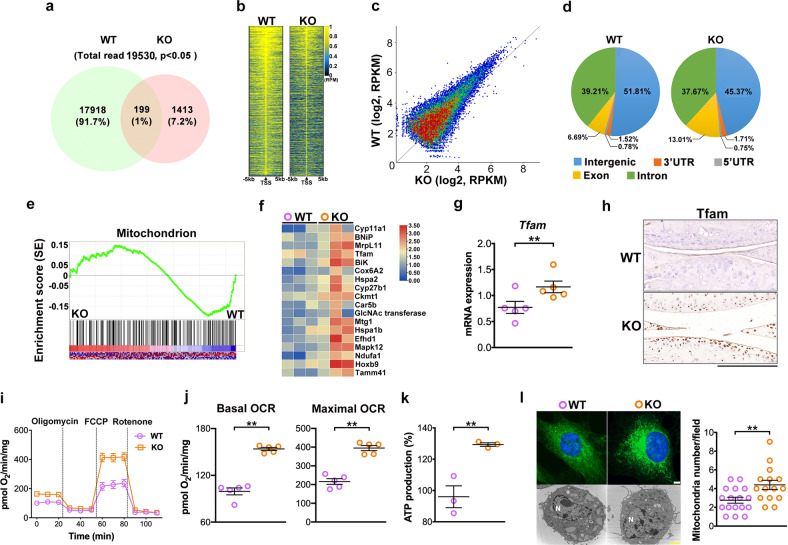
ChIP-seq analyses of H3K27me3 in chondrocytes. Venn diagram showing the overlap of H3K27me3 peaks between UtxKO and WT mice. Total numbers of H3K27me3-enriched binding sites and their relative proportions (%) are indicated. Note that UtxKO mice revealed fewer binding sites compared to WT animals (**a**). Heatmaps of ChIP-seq analyses showing H3K27me3 DNA occupancy within 5 kb upstream and downstream, respectively, of transcriptional start site (TSS) (**b**). Scatter plots shows H3K27me3 ChIP-seq signals distribution in UtxKO and WT (**c**), next to the relative distribution of H3K27me3 occupancy across genomic regions in chondrocytes of WT and UtxKO animals, respectively (**d**). Gene set enrichment analysis of H3K27me3 marks revealed significant enhancement mitochondrion in UtxKO cells (**e**, **f**). Data are calculated from three mice. Tfam mRNA expression (**g**), Tfam immunostaining (**h** scale bar, 10 μm) mitochondrial respiration profiles (**i**), basal and maximal oxygen consumption rates (**j**), ATP production (**k**), and mitochondrial mass as evidenced in MitoTracker Green staining (scale bar, 20 μm) and electron transmission microscopy (scale bar, 200 nm) (**l**) in UtxKO chondrocytes were higher than in WT cells. Data are expressed as mean ± standard errors calculated from three to five mice. *P < 0.05; **P < 0.001.

We conducted genome-wide ChIP-Seq analysis to identify changes in the H3K27me3-binding epigenomic landscape that might have contributed to the improved chondrocyte marker gene expression in UtxKO mice. Of 19531 H3K27me3-binding sites, 91.7% and 7.2% were enriched in genomes of WT and UtxKO animals, respectively, (Fig. 5a). Utx loss globally decreased the H3K27me3 occupancy in the promoters preferably within a 5 kb up- and downstream region of the respective transcriptional start sites (Fig. 5b), which consequently resulted in a higher normalized transcriptional expression (RPKM) (Fig. 5c) and a higher distribution in exon region (Fig. 5d).

Gene set enrichment analyses (Fig. 5e) revealed that Utx deletion was advantageous to mitochondrial activity as the transcription of mitochondrial transcription factor Tfam and electron transfer chain enzymes (Fig. 5f) were increased in UtxKO chondrocytes. Consistently, Tfam mRNA expression (Fig. 5g) and immunoreactivity (Fig. 5h), mitochondrial respiration profiles (Fig. 5i), basal and maximal oxygen consumption rate (Fig. 5j), ATP production (Fig. 5k), and mitochondrial mass as evidenced in MitoTracker Green staining and transmission electron microscopy (Fig. 5l) in UtxKO mice were higher than in WT animals.

### Igf2-mediated Utx loss-induced promotion of cartilage ECM production

**Fig. 6:**
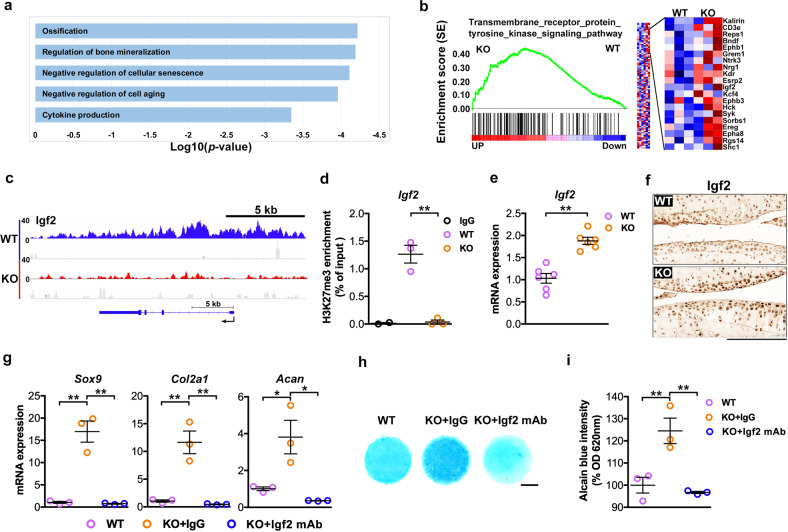
UTX-dependence of H3K27me3-mediated Igf2 transcriptional repression. Selected H3K27me3-enriched GO terms in UtxKO chondrocytes (**a**). Gene set enrichment analysis of H3K27me3 marks revealed significant enhancement a specific pathway in UtxKO cells. Igf2 was selected as differentially regulated and earlier reported H3K27me3-mediated candidates controlling chondrocyte differentiation (enlarged section) (**b**). Gene track illustrating limited H3K27me3 chromatin occupation at regulator loci and promoter regions of Igf2 gene in UtxKO chondrocytes (red) as compared to WT cells (blue) (**c**). This confirmed highly significant differences in the enrichment of H3K27me3 by ChIP-PCR assay (**d**). Increased transcription of Igf2 in UtxKO cells (**e**) and evident Igf2 immunostaining (scale bar, 10 μm) in UtxKO cartilage (**f**). Antibody-mediated blockade of Igf2 suppressed Sox9, Col2a1, and Acan expression (**g**), as well as glycosaminoglycan production (**h**, **i** scale bar, 500 μm), in UtxKO chondrocytes. Cells were incubated in medium with Igf2 antibody or IgG. RT-PCR was conducted upon antibody treatment for 24 h. Micromass for Alcian blue staining was incubated for 7 days. Cell cultures were harvested from three mice and experiments were repeated three times. Data are expressed as mean ± standard errors. *P < 0.05; **P < 0.001.

Pathway ontology analyses also suggested that Utx knockout facilitated cellular processes related to cartilage and skeletal development, including ossification and bone mineralization, as well as enhanced inhibition of cellular senescence (Fig. 6a). From the H3K27me3 repressive signatures, Igf2 was selected for subsequent experiments (Fig. 6b) as it is known to control cartilage development during postnatal bone growth [21] and correlate with human OA [22]. The spatial distribution of H3K27me3-binding sites in the coding sequence of Igf2 (Fig. 6c) and the relative H3K27me3 occupancy at the gene (Fig. 6d) were reduced in UtxKO chondrocytes. Igf2 mRNA expression (Fig. 6e) and immunostaining (Fig. 6f) were consequently increased and expression of Sox9, Col2a1, and Acan (Fig. 6g, IgG control) and glycosaminoglycan synthesis (Fig. 6h, i) were much higher in UtxKO cells than in WT cells. These effects were reversed upon Igf2 antibody treatment. Our data suggest that Igf2 positively stimulated cartilage ECM synthesis.

### PRC2 involved Utx loss-induced H3K27 hypomethylation

**Fig. 7:**
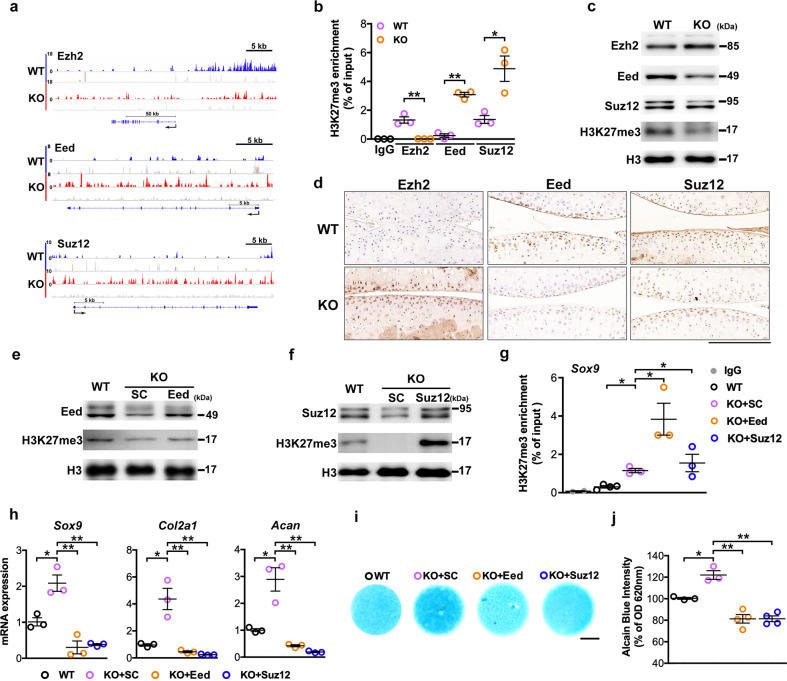
PRC2 core components contributed to Utx depletion-mediated anabolic chondrocytic activity. Gene track illustrating limited H3K27me3 chromatin occupation at regulator loci and promoter regions of Ezh2, Eed, and Suz12 *genes* in UtxKO chondrocytes (red) as compared to WT cells (blue) (**a**). Utx loss repressed the H3K27me3 enrichment at the Ezh2 promoter but increased that at the Eed and Suz12 promoters (**b**). It increased abundance of Ezh2, but reduced that of Eed, Suz12, and H3K27me3 (**c**). Evident Ezh2 immunostaining and weak Eed2 and Suz12 immunoactivity in UtxKO cartilage (**d**), scale bar, 10 μm. Forced Eed or Suz12 expression increased H3K27me3 levels (**e**, **f**) and H3K27me3 occupancy at the Sox9 promoter (**g**). Chondrocytic marker gene expression (**h**) and ECM production (**i**, **j** scale bar, 500 μm) in UtxKO chondrocytes were suppressed by forced expression of both components. Cells were transfected with Eed or Suz12 expression vectors or empty vectors. RT-PCR was conducted upon transfection for 24 h. Micromass for Alcian blue staining were incubated for 7 days. Cell cultures were harvested from three mice and experiments were repeated three times. Data are expressed as mean ± standard errors. *P < 0.05; **P < 0.001.

To understand how a deficiency in methyl histone eraser Utx caused a seemingly contradictory H3K27 hypomethylation, we investigated whether PRC2 function was changed in chondrocytes. Utx loss downregulated the spatial distribution (Fig. 7a) and the enrichment of H3K27me3 (Fig. 7b) at the Ezh2 promoter, whereas they were increased at the Eed and Suz12 promoters. Increased Ezh2 but reduced Eed, Suz12, and H3K27me3 levels were evident in UtxKO cells (Fig. 7c). Consistently, Ezh2 immunostaining was increased and Eed and Suz12 immunoreactivities were repressed in UtxKO cartilage (Fig. 7d).

Forced Eed (Fig. 7e) or Suz12 (Fig. 7f) expression increased H3K27me3 abundance and the H3K27me3 occupancy at the Sox9 promoter, relative to WT cells (Fig. 7g). This subsequently repressed Utx expression of chondrocyte markers (Fig. 7h) and ECM synthesis (Fig. 7i, j) in UtxKO chondrocytes. Experimental results suggested that loss-of-function of Utx dysregulated PRC2 actions, maintaining H3K27 relatively hypomethylated and therefore enhanced chondrocytic marker expression. Low Eed or Suz12 levels appeared to inhibit Ezh2 and its ability to H3K27 trimethylation.

## Discussion

Epigenetic changes occur during OA and histone methylation appear to dysregulate chondrocyte metabolism [23, 24]. Our data now associated expression of histone eraser UTX with OA development in humans and correlated UTX and H3K27 trimethylation in articular cartilage with chondrocyte marker expression in vitro. This was in agreement with the only other study demonstrating high H3K27me3 abundances in human hip osteoarthritic cartilage [14]. We further, for the first time, manipulated Utx activity through RNAi and lentiviral overexpression and established a murine UtxKO model. Our comprehensive study revealed a novel catabolic role of the H3K27me2/3-specific demethylase Utx, in concert with PRC2 core components, in articular chondrocyte homeostasis and the development of OA.

We used Chip-Seq to screen global changes in gene transcription between WT and UtxKO chondrocytes to identify molecular mechanisms protecting cartilage degradation. GO terms already revealed major expressional changes occurring in gene regulation networks related to mitochondrial activity, mineralization, ossification, and senescence. Sox9 is a prominent activator of *Col2a1* transcription, making it one of regulators of the chondrocyte phenotype [25]. To show a Utx-dependent compromised chondrocytic activity, we used key chondrocyte marker genes *Sox9*, *Col2a1,* and *Acan* as readouts, and quantified proteoglycan production as a measure of ECM quality. Using Utx knockdown confirmed anabolic effects on ECM level in vitro and being in line with the observed overall improved relative thickness of the articular cartilage in vivo.

The collective analysis confirmed that Utx deletion promoted mitochondrial biogenesis, including Tfam expression, mitochondrial respiration, and ATP production. Candidates, like Igf2 arising from enrichment scores were then further analyzed to reveal H3K27me3 occupation alteration at its promoter. IGF2 is known to regulate chondrocyte function and cartilage development [21, 22]. To the best of our knowledge, no further information on a direct involvement of Utx in regulating the IGF signaling exists in OA, while IGF2 is known to regulate cartilage development [21]. IGF2 also compromises ECM loss in inflamed chondrocytes and preserves cartilage integrity even in a model of experimental osteoarthritis [26]. This may, partly, explain the promoting effects of UtxKO on ECM synthesis in chondrocytes. IGF signaling also regulates cell proliferation, differentiation, and apoptosis in cartilage [27]. Controlling IGF signaling indirectly through Utx modulation was thus advantageous to articular cartilage integrity.

Our enrichment scores also revealed upregulation of many genes associated with a proper articular chondrocyte function and cartilage integrity, like BMP antagonist Grem1, Ntrk3, Syk, and Shc1 [28–31]. BMP signaling ultimately induced unwanted hypertrophy in articular chondrocytes. Leijten et al. already showed that decreased GREM1 expression in cartilage were correlated with OA [32]. While BMPs may exert pro-hypertrophic actions under certain conditions [33], the upregulation of BMP antagonists may thus explain the overall chondroprotective net effect of Utx loss in vitro and in vivo.

Utx knockout largely improved signs of cartilage degeneration in primary OA in old mice and in two experimental murine models of induced secondary OA development. The biological role of Utx in chondrocyte function remains uncertain. Utx knockdown represses chondrogenic differentiation capacity of human periodontal progenitor cells [34]. Yapp et al. recently studied Utx in chondrogenic differentiation of human mesenchymal stem cells and ECM production in chondrocytes, using Utx inhibitor GSK-J4 [35]. These results hint toward a cartilage-deleterious effect of Utx inhibition, but it is well accepted that the complex TGF-β signaling in adult cartilage in vivo cannot be fully appreciated by in vitro models of chondrogenesis used in that study. Adult articular cartilage is considered a post-mitotic tissue with terminally differentiated chondrocytes [36]. A “phenotypic plasticity” of articular chondrocytes has then been associated with OA [37] in which chondrocytes de-differentiate towards a more fibroblast-like phenotype [38]. This study showed that Utx appeared to play a context-dependent role in the development of OA.

UTX usually removes di- and trimethyl groups on H3K27 to promote target gene activation [13]. Surprisingly, loss of Utx function now activated chondroprotective pathways and mRNA expression of Igf2 and Sox9 in particular. In addition, Utx deletion contraintuitively reduced the H3K27me3 occupation at the promoter loci of these genes. Apparently, other regulatory pathways potentially contributed and UTX loss can indeed enhance the EZH2-induced H3K27 trimethylation [39]. We thus postulated that PRC2 core components participated in the UTX loss-induced H3K27 hypomethylation in chondrocytes. Our data now revealed that upon Utx loss, PRC2 core components Eed and Suz12 appeared to curtail chondrocyte metabolism as restoring Eed or Suz12 resulted in H3K27 hypermethylation and compromised ECM synthesis. Increasing evidence has shown that EZH2 upregulates the production of cartilage catabolic factors [40] and apoptosis program [41] in inflamed human chondrocytes. EED thus appears functionally indispensable for trimethylation of H3K27 by EZH2 [42]. Our study thus revealed a new paradigm in which opposing action of PRC2 components are responsible for UTX loss-mediated H3K27 hypomethylation to maintain proper ECM homeostasis in cartilage. Weak Eed and Suz12 actions blocked EZH2-mediated H3K27 trimethylation, driving UtxKO chondrocytes to produce abundant extracellular matrices.

In mammals, PRC2 core components EZH2 and EZH1 are important for writing trimethylation of H3K27 [43]. PRC2 and H3K27me3 are involved in bivalent control of transcription activation and repression during stem cell fate commitment [44], in line with the earlier discussed plasticity of chondrocytes in OA. To this end, our data are in agreement with studies demonstrating that UTX knockout decreases H3K27 trimethylation to alter mesenchymal stem cells differentiation [45]. In general, polycomb-group proteins together with their target genes control differentiation program in a dynamic manner. Co-localization of PRC2 with H3K27me3 is required to catalyze trimethylation [44]. As polycomb-group proteins regulate gene silencing, repressing transdifferentiation in an H3K27me3-dependent manner [46] and the latter appears to be the link between inflammation and reprogramming of the epigenome, this may—at least partly—explain our findings.

**Fig. 8:**
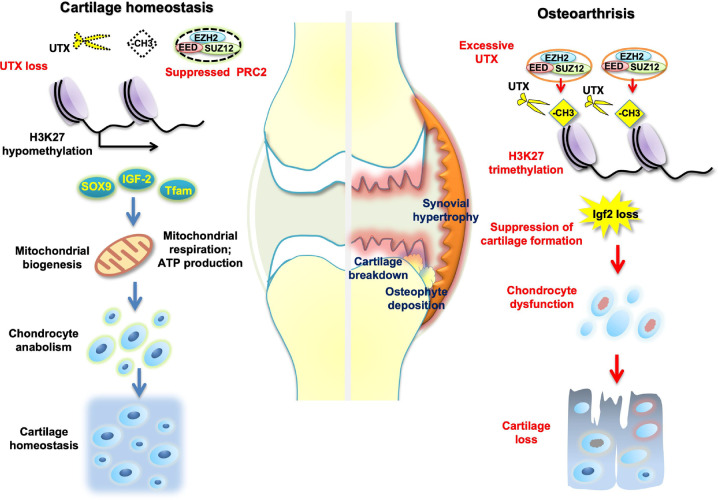
Schematic illustration of how epigenetic changes affect cartilage ECM integrity. Age-dependent changes in articular chondrocytes that are leading to an increased UTX activity (i.e., mimicked by lentiviral overexpression) are, together with histone writer PRC2 core components EED and SUZ12, resulting in an elevated histone methylation state in these cells, consequently dysregulating cartilage homeostasis. Also, this epigenetic signature suppresses SOX9 activity, which is crucial for maintaining ECM integrity. Ultimately, this culminates in the development of OA. In contrast, inhibiting UTX facilitates transcriptional activity at promoter regions of certain cartilage key markers (like e.g., SOX9, Col2a1, ACAN) at least partially through co-suppression of Eed and Suz12. This net stimulation of anabolic factors then aids in maintaining the ECM integrity of the tissue to ensure proper homeostasis.

In conclusion, Utx loss appears to be chondroprotective. However, a seemingly contradictory trimethylation status was observed at the selected gene loci of key chondrocyte markers: loss of histone eraser Utx caused a depletion of H3K27me3 occupation at these domains. To this end, we identified a novel interaction between Utx and PRC2 core complex components. Our current model of the epigenetic regulation of cartilage metabolism is illustrated in Fig. 8; on bivalent loci, PRC2 activity acts in concert with Utx to either stimulate cartilage anabolism or activate phenotypic de-differentiation and ECM deterioration through activating Igf2 signaling. We showed, for the first time, in a multimodal approach using human samples and animal models as well as RNAi intervention that targeted manipulation of Utx activity appears to hold a lot of potential for the development of future anti-OA therapies.

## Materials and methods

### Human knee biopsies

Experimental protocols for evaluating clinical specimens were approved by Chang Gung Medical Foundation Institutional Review Board (IRB Affidavits: 201800451B0). Thirty-four patients who had radiographic signs of end-stage knee OA were included upon informed consent was obtained. During total knee arthroplasty, specimens were harvested from osteoarthritic regions and macroscopic healthy regions in the joint, lateral to the injured site (diagnosed by two orthopedic surgeons).

### Articular chondrocytes

Animal experiments were approved by the IACUC of Kaohsiung Chang Gung Memorial Hospital (IACUC Affidavit: 2018031502). Seven-day-old male C57BL/6 mice were euthanatized, chondrocytes were isolated from knee joints and incubated in DMEM with 10% fetal bovine serum [47]. Micromass of 5 × 10^5^ cells was incubated for 7 days. ECM synthesis was detected using Alcian blue staining, with the stain dissolved with 50 μl of 6 M guanidine hydrochloride and spectrophotometrically quantified at 620 nm [48]. In some experiments, chondrocytes were incubated in medium with 10 ng/ml IGF2 antibody (R&D Systems) or IgG for 1 day.

### RNAi or cDNA transfection

Plasmids encoded Utx, Eed, Suz12 (System Biosciences) or Utx siRNA (Thermo Fisher Scientific), and packaging vectors were transduced into 293T cells. Lentivirus suspensions were prepared using LentiX RT-qPCR Titration Kits (Clontech). Chondrocytes (5 × 10^5^ cells) were incubated in medium with lentivirus suspension (10^8^ infectious units) for 24 h.

### Lentivirus UTX gene transfer into knee joints

Male C57BL/6 mice (12 weeks old) were anesthetized, 10 μl of UTX or mock lentivirus suspension (1 × 10^8^/μl infectious unit) were intra-articularly injected into left knees. At 8 weeks after injection, animals were euthanized, and knee joints with tibiae and femurs were dissected for μCT and histological analysis.

### Chondrocyte-specific UTX knockout mice

Mice carrying Utx flox (Jackson Laboratory) were mated with C57BL/6 mice carrying cartilage-specific collagen type II (Col2a1) gene promoter-driven Cre recombinase mice (Col2a1^cre/+^ C57BL/6; Jackson Laboratory) to breed homozygous Utx knockout mice (Co12a1^Cre/+^-Utx^fl/fl^; UtxKO) and wild-type mice (Col2a1^Cre/−^-Utx^−/−^; WT). Genotypes were confirmed using primers for flox and Cre (Supplemental Table 1) and ABI 7900 Detection System (Applied Biosystems).

### RT-PCR

Reverse transcription templates were prepared from 1 μg total RNA and mixed with TaqMan® Universal PCR Master Mix (Applied Biosystems) and primers for Utx, Sox9, Col2a1, Acan, Igf2, and 18S rRNA (Supplemental Table 1) for PCR reactions. The amplification specificity and calculation of relative mRNA expression were investigated, as previously described [19].

### Histomorphometry of articular cartilage

Decalcified and paraffin-embedded knee joints were cut into sagittal sections, which were further Safranin-O stained. Images of the medial mid-condylar region of proximal tibiae were captured (Panoramic MIDI II, 3D HISTECH Ltd.), spanning anterior-to-posterior tibial areas, and using six random sections from each specimen in increments of 200 μm. The average thickness of total articular cartilage between articular surface and cement line, uncalcified cartilage portion between tidemark and articular surface, and calcified cartilage zone between tidemark and cement line was measured, as well as articular chondrocytes in each field (50 μm^2^) and six fields in each section were counted, as previously described [49, 50].

### Assessment of OA histopathology and immunohistology

Ten sections of knee joints spanning 200 μm were Safranin-O stained. Articular cartilage destruction was evaluated, according to the guideline of Osteoarthritis Research Society International [51]. Synovitis was graded using a 0–3 scoring system, with 0 = normal, 1 = moderate, and 3 = severe degeneration. Immunoreactivity was examined using Utx (ab253183, Abcam), H3K27me3 (ab6002; Abcam), Tfam (SC166965; Santa Cruz), Igf2 (ab9574; Abcam), Ezh2 (ab283273, Abcam), Eed (ab264566, Abcam), Suz12 (ab126577, Abcam), and Super Sensitive™ IHC Detection Systems (BioGenex Laboratories). Immuno-labeled chondrocytes within each field (50 μm^2^) were counted (nine fields in three sections for each specimen).

### Experimental knee OA models

Twelve-week-old male WT and UtxKO mice were anesthetized, medial patellar tendon and meniscotibial ligament in the left knee joints were cut to induce destabilized medial meniscus (DMM)-induced OA, as previously described [20]. For collagenase-induced knee OA, left knees were intra-articularly injected with 1 unit of collagenase (*Clostridium histolyticum*, Sigma-Aldrich) [19]. Eight weeks upon induction, mice were euthanatized, and knee joints were excised.

### Micro-CT analyses

The microstructure of knee joints was evaluated using a Skyscan 1176 μCT scanner (Bruker). The images of 400 scanned sections were reconstructed using SKYSCAN^®^ CT-Analyser software. Osteophyte formation and trabecular morphology of subchondral bone in proximal tibiae were diagnosed [52]. Osteophyte volume (mm^3^), bone mineral density (g/mm^3^), and total bone volume (BV/TV) were quantified using the software.

### Chromatin immunoprecipitation (ChIP) sequencing

H3K27me3 in 5 × 10^6^ cells were immunoprecipitated using H3K27me3 antibodies (ab6002; Abcam) or IgG (Millipore). Chromatin in the IP was extracted, deproteinized, and condensed using Magna ChIP A/G Kits (Millipores). Whole-genome profiles and 2 × 150 paired-end reads were sequenced using Illumina HiSeq4000 (Illumina, Inc.). CLC Genomics Workbench (v10.) and Transcription Factor ChIP-Seq analysis pipeline were utilized for quality control (>20 M reads), trimming (average length of reads <150 bp), mapping (mouse genome build 38/mm10, total mapped reads >20 M), and peak characterization (peak shape score, and p-value). Fold changes of RPM for peak position were analyzed using DESeq software (R package version 1.16.0). Read peaks annotating transcription start site (TSS) within upstream and downstream 5 kb were characterized using gplots R package (v. 2.17.0, https://CRAN.R-project.org/package=gplots). BAM files were also visualized by the Integrative Genomics Viewer (v.2.4.13) and imported to SeqMock (v.1.42.0) with pair distance cutoff 200 bp. Data are available in GEO database (accession number GSE121698). Genome-wide expression profiles and ontology of aligned gene set were verified using gene set enrichment analysis (GSEA) [53] and KEGG database.

### ChIP-PCR

DNA (0.1 ng) in H3K27me3 immunoprecipitants and Cy3-conjugated primers (Applied Biosystems) (Supplemental Table 1) were pipetted to investigate the sequences of Sox9, Igf2, Ezh2, Eed, and Suz12 promoters using PCR protocols. Ct values for serial dilution of DNA were computed for amplification efficiency. H3K27me3 occupancy in promoters was expressed as % input.

### Mitochondrial respiration and ATP production assay

Basal and maximum mitochondrial oxygen consumption rate (OCR) in 2 × 10^5^ cells were quantified using Agilent Seahorse XFp Cell Mito Stress Test Kits and Seahorse XFe Analyzer (Agilent), according to the manufacturer’s manuals. Mitochondrial ATP levels were measured using ATP Assay Kits (Abcam).

### Analysis of mitochondria mass

Mitochondria in 10^2^ chondrocytes in culture slides were stained using MitoTracker™ Green FM (Thermo Fisher Scientific Inc., Waltham, MA, USA) and evaluated using laser confocal microscopy. In some experiments, transmission electron microscopy (Hitachi SU8229 TEM System) was conducted to evaluate mitochondrial ultrastructure. Mitochondria in each field and six fields in each slide were counted.

### Immunoblotting

Proteins of interest in the blots were investigated using Utx (ab253183, Abcam), Ezh2 (ab283273), Eed (ab264566), Suz12 (ab126577), H3K27me3 (ab6002), H3 antibodies (ab1791), and ECL Western Blotting Substrate Kits (ab133408), according to the makers’ manuals.

### Statistical analyses

The data of human OA and healthy specimens were analyzed using Student’s *t* test. The analysis between WT and UtxKO mice was investigated using Wilcoxon test. ANOVA and Bonferroni post hoc test were used to investigate the data collected from three or more groups. A significant difference was defined as *P* values < 0.05.

## Supplementary information

Supplementary Table 1

Figure S1

Figure S2

Reproducibility Checklist

## Data Availability

ChIP-sequencing data are deposited in GEO database (accession number GSE121698). Data related to animal studies, in vitro models, and human specimens are available on request from the corresponding author. Patients’ medical information is not publicly available due to ethics and confidentiality restrictions.

## References

[1] Sharma L (2021). Osteoarthritis of the knee. N. Eng J Med.

[2] Hunter DJ, Bierma-Zwinstra S (2019). Osteoarthritis. Lancet.

[3] Monteagudo S, Lories RJ (2017). Cushioning the cartilage: a canonical Wnt restricting matter. Nat Rev Rheumatol.

[4] Katz JN, Arant KR, Loeser RF (2021). Diagnosis and treatment of hip and knee osteoarthritis: a review. JAMA.

[5] Kim JH, Jeon J, Shin M, Won Y, Lee M, Kwak JS (2014). Regulation of the catabolic cascade in osteoarthritis by the zinc-ZIP8-MTF1 axis. Cell.

[6] Sen P, Shah PP, Nativio R, Berger SL (2016). Epigenetic mechanisms of longevity and aging. Cell.

[7] Fang D, Gan H, Lee JH, Han J, Wang Z, Riester SM (2016). The histone H3.3K36M mutation reprograms the epigenome of chondroblastomas. Science.

[8] Hata K, Takashima R, Amano K, Ono K, Nakanishi M, Yoshida M (2013). Arid5b facilitates chondrogenesis by recruiting the histone demethylase Phf2 to Sox9-regulated genes. Nat Commun.

[9] Monteagudo S, Cornelis FMF, Aznar-Lopez C, Yibmantasiri P, Guns LA, Carmeliet P (2017). DOT1L safeguards cartilage homeostasis and protects against osteoarthritis. Nat Commun.

[10] Cornelis FMF, de Roover A, Storms L, Hens A, Lories RJ, Monteagudo S (2019). Increased susceptibility to develop spontaneous and post-traumatic osteoarthritis in Dot1l-deficient mice. Osteoarthr Cartil.

[11] Zhang M, Lu Q, Egan B, Zhong XB, Brandt K, Wang J (2016). Epigenetically mediated spontaneous reduction of NFAT1 expression causes imbalanced metabolic activities of articular chondrocytes in aged mice. Osteoarthr Cartil.

[12] Wang SP, Tang Z, Chen CW, Shimada M, Koche RP, Wang LH (2017). UTX-MLL4-p300 transcriptional regulatory network coordinately shapes active enhancer landscapes for eliciting transcription. Mol Cell.

[13] Tran N, Broun A, Ge K (2020). Lysine demethylase KDM6A in differentiation, development, and cancer. Mol Cell Biol.

[14] Kim KI, Park YS, Im GI (2013). Changes in the epigenetic status of the SOX-9 promoter in human osteoarthritic cartilage. J Bone Min Res.

[15] Lui JC, Garrison P, Nguyen Q, Ad M, Keembiyehetty C, Chen W (2016). EZH1 and EZH2 promote skeletal growth by repressing inhibitors of chondrocyte proliferation and hypertrophy. Nat Commun.

[16] Du X, Chen Y, Zhang Q, Lin J, Yu Y, Pan Z (2020). Ezh2 ameliorates osteoarthritis by activating TNFSF13B. J Bone Min Res.

[17] Mirzamohammadi F, Papaioannou G, Inloes JB, Rankin EB, Xie H, Schipani E (2016). Polycomb repressive complex 2 regulates skeletal growth by suppressing Wnt and TGF-β signalling. Nat Commun.

[18] Wang C, Lee JE, Cho YW, Xiao Y, Jin Q, Liu C (2012). UTX regulates mesoderm differentiation of embryonic stem cells independent of H3K27 demethylase activity. Proc Natl Acad Soc USA.

[19] Ko JY, Sun YC, Li WC, Wang FS (2016). Chaperonin 60 regulation of SOX9 ubiquitination mitigates the development of knee osteoarthritis. J Mol Med.

[20] Lorenz J, Grässel S (2014). Experimental osteoarthritis models in mice. Methods Mol Biol.

[21] Uchimura T, Hollander JM, Nakamura DS, Liu Z, Rosen CJ, Georgakoudi I (2017). An essential role for IGF2 in cartilage development and glucose metabolism during postnatal long bone growth. Development.

[22] Timur UT, Jahr H, Anderson J, Green DC, Emans PJ, Smagul A (2021). Identification of tissue-dependent proteins in knee OA synovial fluid. Osteoarthr Cartil.

[23] McCulloch K, Litherland GJ, Rai TS (2017). Cellular senescence in osteoarthritis pathology. Aging Cell.

[24] Frank-Bertoncelj M, Trenkmann M, Klein K, Karouzakis E, Rehrauer H, Bratus A (2017). Epigenetically-driven anatomical diversity of synovial fibroblasts guides joint-specific fibroblasts functions. Nat Commun.

[25] Yasuda H, Oh CD, Chen D, de Crombrugghe B, Kim JH (2017). A novel regulatory mechanism of type II collagen expression via a SOX9-dependent enhancer in intron 6. J Biol Chem.

[26] Uchimura T, Foote AT, Smith EL, Matzkin EG, Zeng L (2015). Insulin-like growth factor II (IGF-II) inhibits IL-1β-induced cartilage matrix loss and promotes cartilage integrity in experimental osteoarthritis. J Cell Biochem.

[27] Zhang L, Smith DW, Gardiner BS, Grodzinsky AJ (2013). Modeling the insulin-like growth factor system in articular cartilage. PLoS ONE.

[28] Chang SH, Mori D, Kobayashi H, Mori Y, Nakamoto H, Okada K (2019). Excessive mechanical loading promotes osteoarthritis through the gremlin-1-NF-κB pathway. Nat Commun.

[29] Jiang Y, Tuan RS (2019). Role of NGF-TrkA signaling in calcification of articular chondrocytes. FASEB J.

[30] Nasi S, So A, Combes C, Daudon M, Busso N (2016). Interleukin-6 and chondrocyte mineralisation act in tandem to promote experimental osteoarthritis. Ann Rheum Dis.

[31] Schulze-Tanzil G, Mobasheri A, de Souza P, John T, Shakibaei M (2004). Loss of chondrogenic potential in dedifferentiated chondrocytes correlates with deficient Shc-Erk interaction and apoptosis. Osteoarthr Cartil.

[32] Leijten JC, Bos SD, Landman EB, Georgi N, Jahr H, Meulenbelt I (2013). GREM1, FRZB and DKK1 mRNA levels correlate with osteoarthritis and are regulated by osteoarthritis-associated factors. Arthritis Res Ther.

[33] Deng ZH, Li YS, Gao X, Lei GH, Huard J (2018). Bone morphogenetic proteins for articular cartilage regeneration. Osteoarthr Cartil.

[34] Wang P, Li Y, Meng T, Zhang J, Wei Y, Meng Z (2018). KDM6A promotes chondrogenic differentiation of periodontal ligament stem cells by demethylation of SOX9. Cell Prolif.

[35] Yapp C, Carr AJ, Price A, Oppermann U, Snelling SJ (2016). H3K27me3 demethylases regulate in vitro chondrogenesis and chondrocyte activity in osteoarthritis. Arthritis Res Ther.

[36] Dunn SL, Soul J, Anand S, Schwartz JM, Boot-Handford RP, Hardingham TE (2016). Gene expression changes in damaged osteoarthritic cartilage identify a signature of non-chondrogenic and mechanical responses. Osteoarthr Cartil.

[37] Vincent TL, Wann AKT (2019). Mechanoadaptation: articular cartilage through thick and thin. J Physiol.

[38] Charlier E, Deroyer C, Ciregia F, Malaise O, Neuville S, Plener Z (2019). Chondrocyte differentiation and osteoarthritis (OA). Biochem Pharm.

[39] Ler LD, Ghosh S, Chai X, Thike AA, Heng HL, Siew EY (2017). Loss of tumor suppressor KDM6A amplifies PRC2-regulated transcriptional repression in bladder cancer and can be targeted through inhibition of EZH2. Sci Transl Med.

[40] Allas L, Brochard S, Rochoux Q, Ribet J, Dujarrier C, Veyssiere A (2020). EZH2 inhibition reduces cartilage loss and functional impairment related to osteoarthritis. Sci Rep..

[41] Wang J, Wang X, Ding X, Huang T, Song D, Tao H (2021). EZH2 is associated with cartilage degeneration in osteoarthritis by promoting SDC1 expression via histone methylation of the microRNA-138 promoter. Lab Invest.

[42] Moody JD, Levy S, Mathieu J, Xing Y, Kim W, Dong C (2017). First critical repressive H3K27me3 marks in embryonic stem cells identified using designed protein inhibitor. Proc Natl Acad Sci USA.

[43] Lee MG, Villa R, Trojer P, Norman J, Yan KP, Reinberg D (2007). Demethylation of H3K27 regulates polycomb recruitment and H2A ubiquitination. Science.

[44] Di Croce L, Helin K (2013). Transcriptional regulation by Polycomb group proteins. Nat Struct Mol Biol.

[45] Shan Y, Liang Z, Xing Q, Zhang T, Wang B, Tian S (2017). PRC2 specifies ectoderm lineages and maintains pluripotency in primed but naïve ESCs. Nat Commun.

[46] De Santa F, Totaro MG, Prosperini E, Notarbartolo S, Testa G, Natoli G (2007). The histone H3 lysine-27 demethylase Jmjd3 links inflammation to inhibition of polycomb-mediated gene silencing. Cell.

[47] Gosset M, Berenbaum F, Thirion S, Jacques C (2008). Primary culture and phenotyping of murine chondrocytes. Nat Proc.

[48] Wang X, Cornelis FMF, Lories RJ, Monteagudo S (2019). Exostosin-1 enhances canonical Wnt signaling activity during chondrogenic differentiation. Osteoarthr Cartil.

[49] Jia H, Ma X, Tong W, Doyran B, Sun Z, Wang L (2016). EGFR signaling is critical for maintaining the superficial layer of articular cartilage and preventing osteoarthritis initiation. Proc Nat Acad Sci USA.

[50] Nomura M, Sakitani N, Iwasawa I, Kohara Y, Takano S, Wakimoto Y (2017). Thinning of articular cartilage after joint unloading or immobilization. An experimental investigation of the pathogenesis in mice. Osteoarthr Cartil.

[51] Glasson SS, Chambers MG, Van Den Berg WB, Little CB (2010). The OARSI histopathology initiative-recommendations for histological assessment of osteoarthritis in the mouse. Osteoarthr Cartil.

[52] Lian WS, Ko JY, Wu RW, Sun YC, Chen YS, Wu SL (2018). MicroRNA-128a represses chondrocyte autophagy and exacerbates knee osteoarthritis by disrupting Atg12. Cell Death Dis.

[53] Subramanian A, Tamayo P, Mootha VK, Mukherjee S, Ebert BL, Gillette MA (2005). Gene set enrichment analysis: a knowledge-based approach for interpreting genome-wide expression profiles. Proc Natl Acad Sci USA.

