# Supplementary material for: Histone H3K27 demethylase UTX compromises articular chondrocyte anabolism and aggravates osteoarthritic degeneration
Source: Cell Death Dis. 2022 Jun 8;13(6):538. doi: 10.1038/s41419-022-04985-5 (PMC9178009; doi:10.1038/s41419-022-04985-5)
Supplement: Supplementary file 1 — Supplementary Table 1 [file 41419_2022_4985_MOESM1_ESM.docx]

**Supplementary Table 1**

**Primers for RT-quantitative PCR and ChIP-PCR assays**

| **Primers for RT-quantitative PCR assays of human specimens** | | |
| --- | --- | --- |
| *UTX* | Forward: 5’-CTTCAGCCATTTCAACAGCA-3’  Reverse: 5’-GCTGAGCTGGGGTATATGGA-3’ |  |
| 18S *rRNA* | Forward: 5’-GTAACCCGTTGAACCCCATT-3’  Reverse: 5’-CCATCCAATCGGTAGTAGCG-3’ |  |
| **Primers for PCR assay of genotypes of UtxKO mice** | |  |
| Flox | Forward: 5’-TGAACGCTTACGGAAC-3’  Reverse: 5’-AAATCATGCTGGAACCTAGAAC-3’ |  |
| Cre | Forward: 5’-AGGTGACGTAATTCAGG-3’  Reverse: 5’-CAATTGCTCATATGGACATGTAC-3’ |  |
| **Primers for RT-quantitative PCR assays of mouse specimens** | | |
| *Utx* (Exon 24) | Forward: 5’-CATCAAGAAAATAACAACTTCTGTTCAGT-3’  Reverse: 5’-AAAACACCCCAGTAGCCTTCAG-3’ |  |
| *Col2a1* | Forward: 5’- ACTTTCCTCCGTCTACTG-3’  Reverse: 5’- CCTCATCTCTACATCATTGG-3’ |  |
| *Acan* | Forward: 5’- CGAGTCAACAGCATCTACC-3’  Reverse: 5’-GAGTCATTGGAGCGAAGG-3’ |  |
| *Sox9* | Forward: 5’-GAACGAGAGCGAGAAGAG-3’  Reverse: 5’-CTTGAAGATAGCATTAGGAGAG-3’ |  |
| *Igf-2* | Forward: 5’-AGTCGATGTTGGTGCTTCTC-3’  Reverse: 5’-GACAAACTGAAGCGTGTCAAC-3’ |  |
| *Wnt10a* | Forward: 5’-GAGAGAGTGCTTTCGCCTAC-3’  Reverse: 5’-ACCGCAAGCCTTCAGTTTA-3’ |  |
| *Wnt4* | Forward: 5’-CCGGCTTTGTGGCTTTATTC-3’  Reverse: 5’-AGTGTAGAGGAGGGAGAAGAC-3’ |  |
| *Wnt7a* | Forward: 5’-TTACTGCTGGCACCGTCTGATGTG-3’  Reverse: 5’-TGTCTGCTTCATCCTGAGAAATAATCTCC-3’ |  |
| *Dkk1* | Forward: 5’- CCAAACCTTCTCCTGTCAGTAA-3’  Reverse: 5’-TCAGCACTCAGAACTTCATCTC-3’ |  |
| *sFRP1* | Forward: 5’- CAAGCCTCTTGCCACTAGAA -3’  Reverse: 5’- CTGTAGTCAGCGTCTCCTTAAC -3’ |  |
| 18S *rRNA* | Forward: 5’-GTAACCCGTTGAACCCCATT-3’  Reverse: 5’- CCATCCAATCGGTAGTAGCG-3’ |  |
| **Primers for ChIP-PCR** | | |
| *Sox9* | Forward: 5’-TCAACCCCGGAGTAGTTTG-3’  Reverse: 5’-GGGGAATCAATGAAAACCAA-3’ |  |
| *Igf-2* | Forward: 5’-AACAAGGTGCACCGGTTTAG-3’  Reverse: 5’-CCCACCCAGTATGAGCAGAT-3’ |  |
| *Wnt10a* | Forward: 5’-CACGATGGGGAGAGTTAGGA-3’  Reverse: 5’-CTGCAGGCTGATGACACACT-3’ |  |
