# Supplementary figures and images for: Histone H3K27 demethylase UTX compromises articular chondrocyte anabolism and aggravates osteoarthritic degeneration
Source: Cell Death Dis. 2022 Jun 8;13(6):538. doi: 10.1038/s41419-022-04985-5 (PMC9178009; doi:10.1038/s41419-022-04985-5)

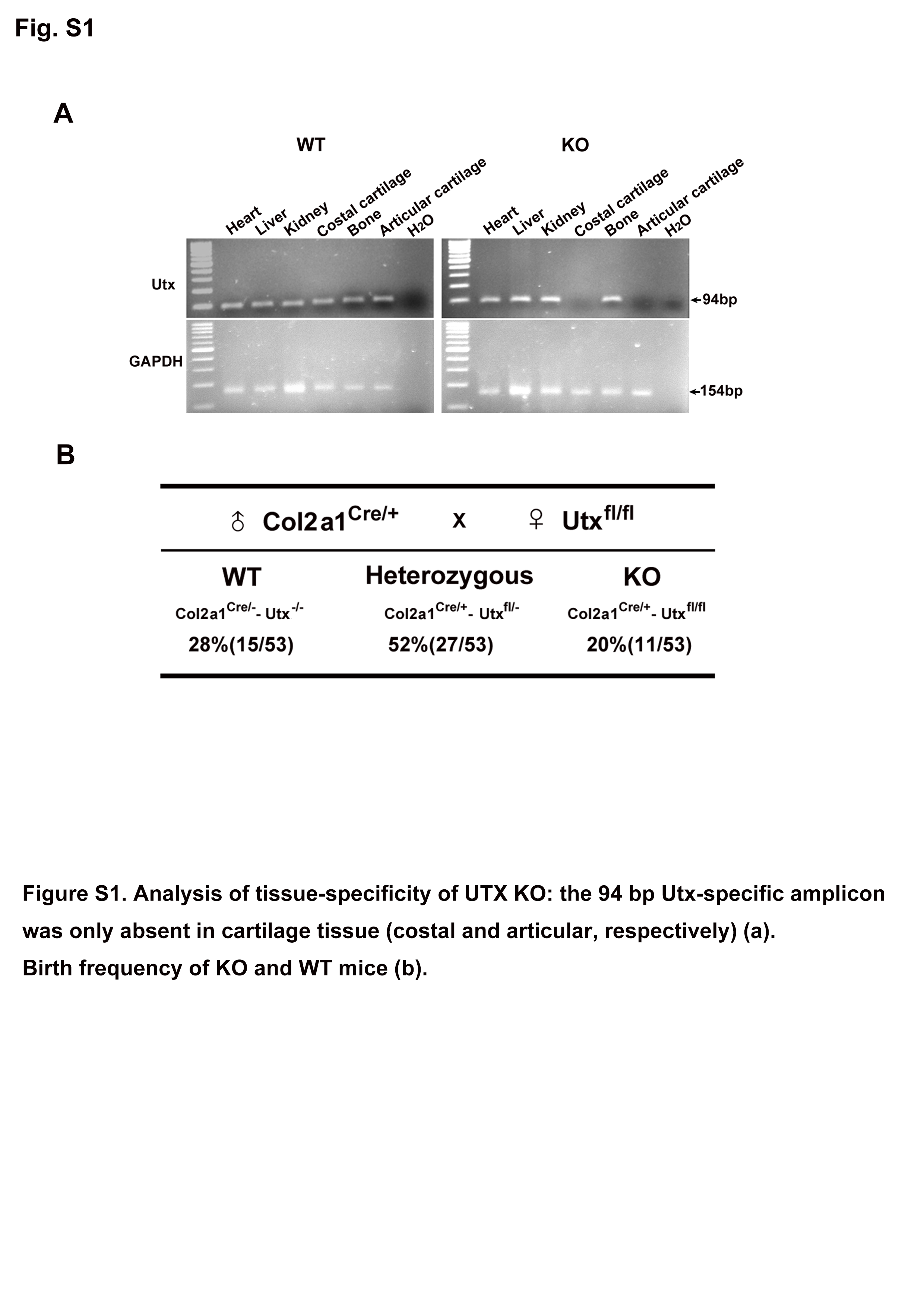

Supplement: Supplementary file 2 — Figure S1 [file 41419_2022_4985_MOESM2_ESM.tif]

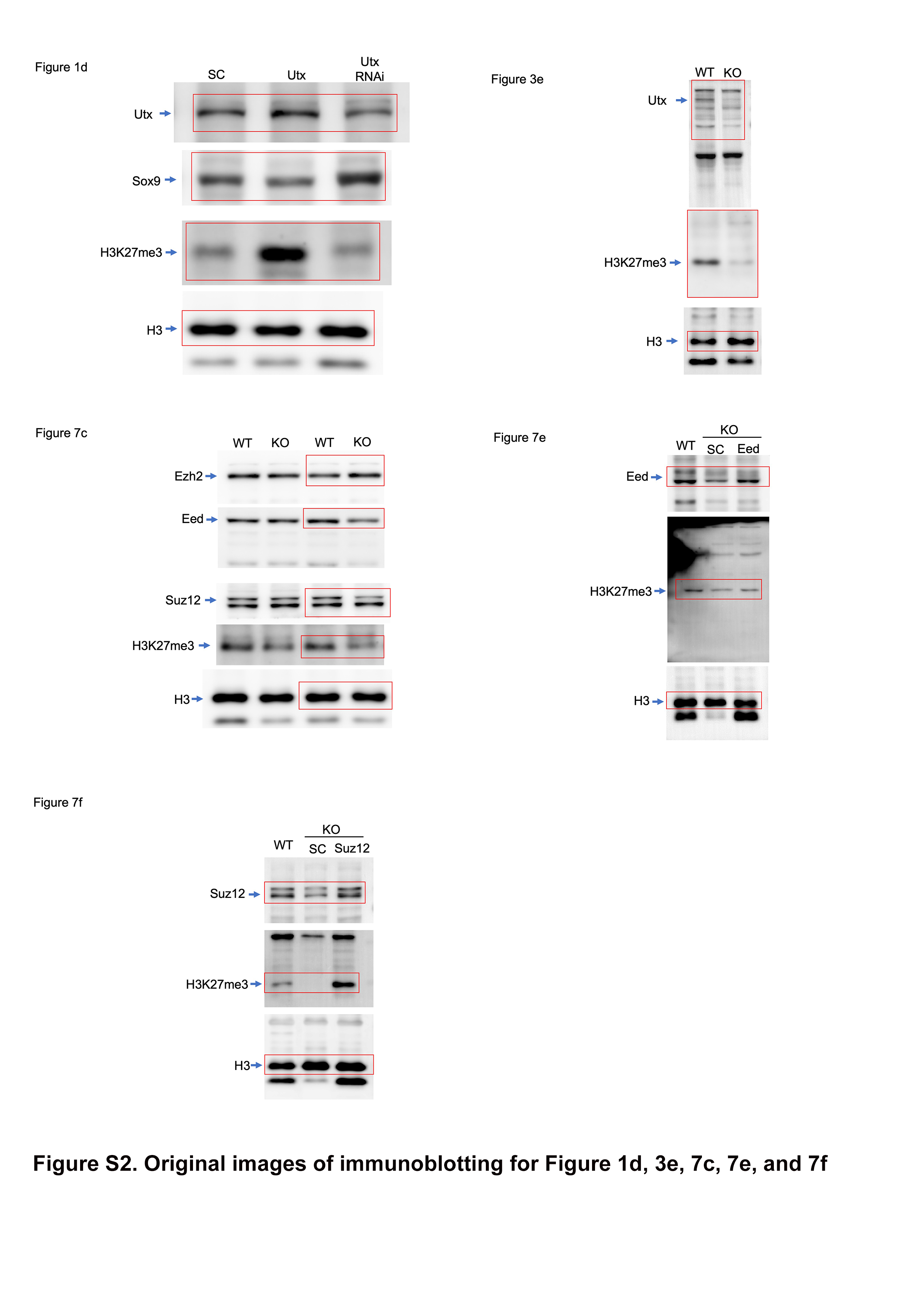

Supplement: Supplementary file 3 — Figure S2 [file 41419_2022_4985_MOESM3_ESM.tif]
